# Foliar Endophytic Fungi from the Endangered Eastern Mountain Avens (*Geum peckii*, *Rosaceae*) in Canada

**DOI:** 10.3390/plants10051026

**Published:** 2021-05-20

**Authors:** Sarah J. Adams, Brent M. Robicheau, Diane LaRue, Robin D. Browne, Allison K. Walker

**Affiliations:** 1 Department of Biology, Acadia University, Wolfville, NS B4P 2R6, Canada; sarahadams@acadiau.ca; 2 Department of Biology, Dalhousie University, Halifax, NS B3H 4R2, Canada; brobicheau@dal.ca; 3 Mersey Tobeatic Research Institute, Mount Merritt, NS B0T 1B0, Canada; laruedi@gmail.com; 4 K.C. Irving Environmental Science Center, Acadia University, Wolfville, NS B4P 2R6, Canada; robin.browne@acadiau.ca

**Keywords:** foliar fungal endophytes, Eastern Mountain Avens, mycobiome, plant conservation, *Rosaceae*

## Abstract

Eastern Mountain Avens (*Geum peckii* Pursh, *Rosaceae*) is a globally rare and endangered perennial plant found only at two coastal bogs within Digby County (Nova Scotia, Canada) and at several alpine sites in the White Mountains of New Hampshire (USA). In Canada, the *G. peckii* population has declined over the past forty years due in part to habitat degradation. We investigated the culturable foliar fungi present in *G. peckii* leaves at five locations with varying degrees of human impact within this plant species’ Canadian range. Fungal identifications were made using *ITS* rDNA barcoding of axenic fungal cultures isolated from leaf tissue. Differences in foliar fungal communities among sites were documented, with a predominance of Gnomoniaceae (Class: Sordariomycetes, Phylum: Ascomycota). Habitats with more human impact showed lower endophytic diversities (10–16 species) compared to the pristine habitat (27 species). Intriguingly, several fungi may represent previously unknown taxa. Our work represents a significant step towards understanding *G. peckii*’s mycobiome and provides relevant data to inform conservation of this rare and endangered plant.

## 1. Introduction

The majority of land plants interact with fungi either as endophytes, which live asymptomatically within plant tissues showing no signs of disease, or as arbuscular or ectomycorrhizae living in symbiosis with plant roots [1,2]. Endophytes can be symbionts, latent decomposers that will eventually aid in the rapid breakdown of the plant upon its senescence, or latent pathogens that will only show symptoms when the host plant experiences certain types of physiological stress [3,4]. Individual endophytic species can play one or all three of these roles during its time within a host, or it may simply remain inert until it re-enters the environment upon the plant’s death and decomposition [3]. Endophytic fungi can be beneficial; some improve plant health and confer resilience to biotic and abiotic stressors, such as through the production of anti-herbivory or anti-microbial toxins, or by the acquisition and biosynthesis of compounds for nutrition [1,4,5]. Overall, these factors can help host plants adapt to changes in the surrounding habitat or environment [4]. Since endophyte-plant interactions can represent an important facet of a plant’s lifecycle—and perhaps even its overall success and survival—it is crucial to understand the endophytic fungi of rare and endangered plants as this knowledge can inform restoration goals [6,7]. For example, exposure to select endophytes may aid in the growth and development of host tissue [3,7] and pest protection. For example, the foliar endophyte of spruce, *Phialocephala scopiformis*, produces rugulosin, which inhibits the feeding of the spruce budworm, *Choristoneura fumiferana*. Consequently, the Canadian forestry industry inoculates spruce seedlings with *P*. *scopiformis* to improve host health [8].

**Figure 1:**
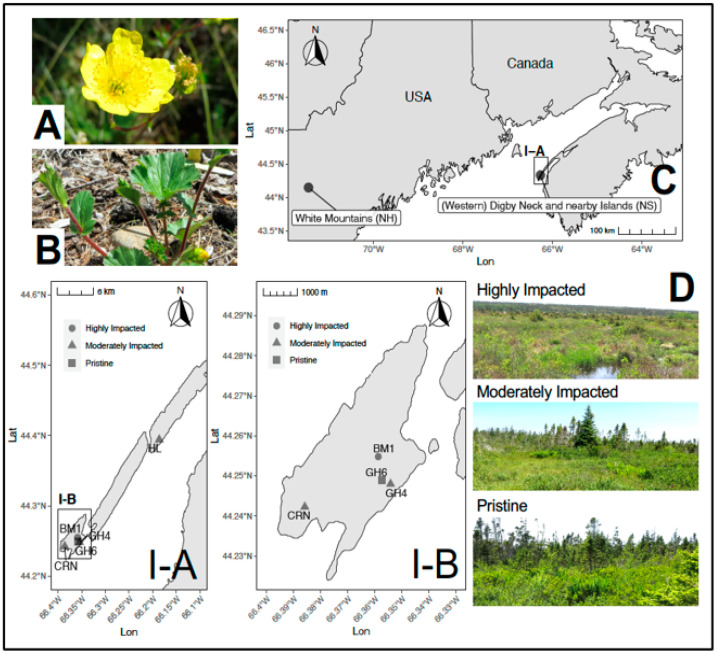
Study species and study sites. (**A**) *Geum peckii* flower (average leaf diameter = 2.5–3.5 cm) and (**B**) leaves (average width = 5–10 cm). (**C**) Locations of the global populations of *Geum peckii*. Locations sampled herein from the Nova Scotian populations are shown in map inset (**I**–**A**) (HL = Western Digby Neck, NS) and inset (**I**–**B**) All other sites = Brier Island, NS). (**D**) Examples of foliage at sites with different levels of habitat impact (moderately impact site shown is CRN).

The Eastern Mountain Avens, *Geum peckii*, is a perennial herbaceous plant (Figure 1a,b) that is globally rare and is only found in the coastal sea level bogs of Brier Island and Digby Neck in Digby County (Nova Scotia, Canada) and is considered to be endemic to the alpine environments of the White Mountains in New Hampshire (United States; Figure 1c) [9,10]. Though separated by approximately 400 km, the New Hampshire and Nova Scotian populations show little genetic drift from each other based on an allele frequency change value of 0.0462 [11]. This suggests that this plant species may have spread from New Hampshire to Nova Scotia after the retreat of the last ice age over 12,000 years ago and before sea level rose, inundating the Georgian Bank and filling the Bay of Fundy [9,12]. Modern populations may therefore be the remnants of a once—larger North American range [13]. *G*. *peckii* exhibits clonal growth by producing rosettes and the expansion of rhizomes [9,10,13,14]. Low germination success was observed in wild population of *G*. *peckii* and other *Geum* species [10,14]. Although *G*. *peckii* readily expands clonally when conditions are suitable, greenhouse and field tests have shown that *G*. *peckii* seeds do not germinate in the mineral soil of Brier Island and long seed storage periods below 0 °C significantly reduce seed viability [15]. However, greenhouse trials have shown almost 100% success when rhizomes with or without rosettes are transplanted into peat amended mineral soil from Brier Island [13]. Field trials with plants propagated by tissue culture and transplanted into a new location with suitable habitat demonstrated almost 100% survival [15].

Interestingly, *Geum* species can produce over 200 biologically active compounds, including antifungal methanols and antioxidant phenols [16]. The presence of these compounds aids in the survival of the plant in adverse habitat conditions and inhibits the ability of some fungi to colonise the plant [16,17,18,19].

We examined plants from the declining Nova Scotian population from several locations (see sites in Figure 1 inset I–A and I–B). Factors contributing to habitat degradation at some sites include: the former use of bog land as pasture, bog drainage for a failed agricultural enterprise, presence of a large Herring gull (*Larus argentatus*) colony, as well as ATV trails that are now unused [9]. Prior to our study, the endophytes of *G*. *peckii* had not been examined.

Using several sites with differing degrees of human impact, we compared the foliar endophytic fungi present in *G*. *peckii*. Our two aims were: a) to first characterise endophytic species present in *G*. *peckii*, and then b) to investigate whether a relationship between immediate habitat conditions and endophyte assemblages is evident. Our approach considered two sampling times (June and July) and two nutrient media types (MEA and ½ MS + A). Our dataset provides both valuable insight into the endophytes that can reside within *G*. *peckii* in connection with habitat differences, as well as a list of fungi that will be of interest for future restoration and conservation applications. From a broader perspective, our methodology may also be of interest to the general study of endangered plant mycobiota.

## 2. Results

Using ITS rDNA barcoding we identified 327 endophytic isolates from 844 leaf discs collected from five study sites across two months, representing 51 fungi (Table 1). Of these, 29 could only be identified to genus based only on the closest type sequence matches in NCBI GenBank showing pair-wise identity scores of >97% for the isolates in question; see Supplementary Table S1, and Figures S1–S6. Several fungi recovered may represent species that are unrepresented in the current GenBank database.

It is important to note that the sampling effort differed by month due to i) the number of sites that could be sampled, as outlined by the scientific permit issued by the Nova Scotia Department of Natural Resources, and ii) during June sampling, the oldest + youngest leaves were collected from each plant to determine if there was a difference in their fungal community. Since no difference was observed between old and young leaves, this procedure was not done in July to avoid oversampling this endangered plant species; only youngest leaves were collected in July. Consequently, the total number of leaf discs collected from each site varied: BM1 = 250 (148 in June + 102 in July), GH4 = 112 (July only), CRN = 146 (116 in June + 74 in July), HL = 102 (July only), GH6 = 234 (130 in June + 104 in July). As a result, of uneven sampling, the differing numbers of leaf discs per site hampers in depth comparisons of the fungal communities present at each location. Nevertheless, these data provide a snapshot of total culturable foliar fungal endophyte richness. Fungal isolation from the leaf discs varied by site 74% (CRN) to 98% (GH4) and media type 62–96% (MEA) and 87–100% (1/2 MS+A). Though specifics on isolation success and total number of fungal isolates per disc were not recorded, the emergence of more than one endophyte was observed for the majority of discs.

The total number of fungi observed by site (Table 1) was: GH6 (pristine site) = 27, BM1 (highly impacted) = 16, CRN (moderately impacted site) = 10, GH4 (moderately impacted site) = 14, and HL (moderately impacted site) = 16. Of the 51 taxa observed, only two species, *Cryptodiaporthe aubertii* and *Plagiostoma lugubre*, were found at all five sites (Table 1). Conversely, 38 fungi were each only isolated from a single site, four species were isolated from only two sites, one species was isolated from only three sites, and six species were isolated from only four sites (Table 1). Only six fungi were isolated during both months; however, this trend is likely affected to a great extent by sampling more individuals in July than in June. We found a difference in the fungi recovered depending on isolation media used.

**Figure 2:**
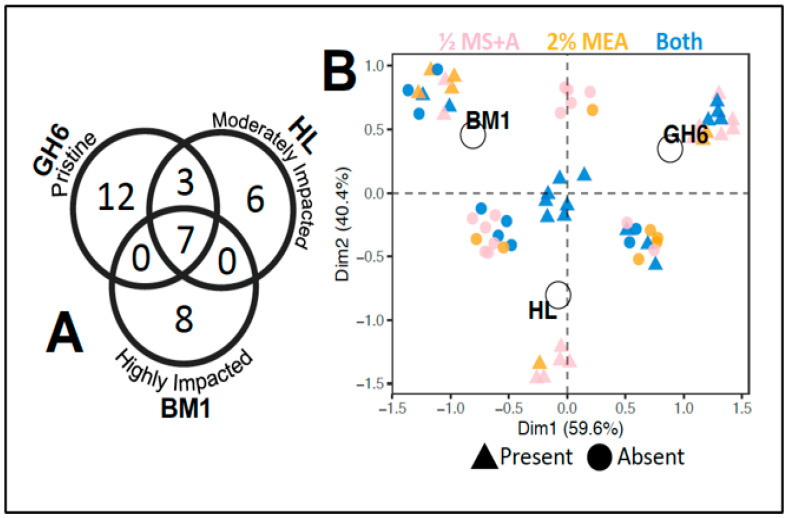
Venn diagram (**A**) and MCA (**B**) comparing the fungal communities reported for BM1 (highly impacted), HL (moderately impacted) and GH6 (pristine) and the media type used for fungal isolations. Please note that only data from July sampling were used in Figure 2.

In an effort to distil site trends related to human impact, multivariate analysis was conducted on a subset of our data. For this analysis only data for sites GH6, HL, and BM1 from July were compared, as these had approximately equivalent numbers of leaf discs (for GH6 n = 104, for HL n = 102, and for BM1 n = 102). The overlap in fungi for just these three sites is shown in the Venn diagram presented in Figure 2a. Overall, seven fungi were shared by all three sites, three fungi were shared between GH6 (pristine) and HL (moderately impacted), while no fungi were shared between GH6 (pristine) and BM1 (highly impacted) nor HL (moderately impacted) and BM1 (highly impacted; Figure 2a). In addition to observing an overlap in species composition, a multiple correspondence analysis (MCA) was used to assess presence/absence trends. MCA results are shown in Figure 2b. For the MCA, Dimension 2 (Dim2) captured 40.4% of the data and showed that the fungi present at HL differ from those of GH6 or BM1 (Figure 2b). Interestingly, HL on Digby Neck was the only site geographically separated from our other sites sampled (see Figure 1 inset I–A). The MCA results suggest that geographic separation between plants may have partially played a role in shaping endophyte assemblages among these three sites. Of the species unique to HL, nearly all fungi were isolated on ½ MS + A media. The remaining 59.6% of the dataset was captured by Dimension 1 (Dim1) of the MCA and showed a separation of the sites by human impact (Figure 2b). Fungi that were unique to GH6 were isolated on ½ MS + A media or both media types, while fungi unique to site BM1 were isolated on 2% MEA or both media types. The seven fungi that were isolated from all sites (GH6, HL, & BM1) in July were found on both media types (Figure 2).

## 3. Discussion

Our results uncovered for the first time a large fraction of the culturable fungi associated with *Geum peckii*. We observed 51 ascomycete fungi from the classes Sordariomycetes, Leotiomycetes, Dothideomycetes and Eurotiomycetes. We observed foliar endophytic richness was higher at the pristine site relative to impacted sites. When attempting to correct for sampling bias, this trend was still upheld, suggesting that along the range of human site impacts sampled, the endophytic richness changes and may act in response to environmental conditions. Further work needs to be done using additional loci for more precise identifications of some fungal specimens to better elucidate their ecological roles. In the discussion that follows, we will review not only the trends in the fungi observed, but also past studies of Sordariomycetes, the taxonomic group most frequently recovered in our samples.

### 3.1. Trends in the Endophytic Fungi from Geum peckii

Previous work has shown that a plant species living across a landscape will share a common core fungal assemblage [20,21,22]. With this in mind, one would expect that all sample sites would to some degree have similar fungal diversities that differ in species due to the limitations of spore dispersal and ecological niches present at each site, while some fungal species would be found regardless of site type [20,22]. There were seven species that were observed at all three sites (i.e., for the sites that had similar sampling intensity). These fungi may be part of a fungal community that is ‘common’ to the bog ecosystems in Digby Neck (NS), and whose presence could be shaped by either a) similar ecological niches being present at all sites, and/or b) these fungal species are associates of *G*. *peckii*. Although it is difficult to tease apart the potential influence of these two scenarios, the notion of fungi being associated with the mycobiome of *G*. *peckii* is of some merit. For instance, *Cryptodiaporthe aubertii*, which was found at all five sites observed during our study, was also found in aseptically grown *G*. *peckii* transplants, which were out planted to a comparable bog habitat on Digby Neck at which *G*. *peckii* had not been observed previously [15,23]. Despite the possibility of *G*. *peckii* having a core set of fungal endophytes, our study found that disturbed sites had lower fungal richness than the pristine site. Due to the human site impacts which have occurred, impacted sites display altered plant communities, plant health, and available ecological niches, compared to the pristine site. These factors are known to impact fungal richness present within a habitat [20,21,22]. In particular, plant communities at the disturbed sites have also been impacted by alterations in their water levels and nutrient deposition, which in turn, have altered the plant species present, their abundance and their health. Others have shown that plants that are healthier are better able to resist fungal colonisation by pathogens [20]. Weakened plant communities are less diverse and are more likely to host fast growing, generalist fungi [21,22]. The majority of the species observed during our study (38 of 51) were documented only once, which is a common finding of endophytic work from temperate climates investigating a single host species [3]. These fungi often have generalist ecologies and may represent the fastest growing species in culture [3]. Our small study size (844 leaf discs across five sites) further indicates that only a small portion of the foliar fungal community of *G*. *peckii* may have been observed during this study.

Our MCA analysis (Figure 2b) also showed that all three of the similarly sampled sites cluster separately based on species composition, suggesting that geography and/or habitat status may be at play in shaping endophytic fungal assemblages. As indicated earlier, site HL is located approximately 20 km northeast of the BM1 and GH6 sites. Although this geographical distance may be enough to alter the fungal assemblages [21], it is important to note that the degradation at BM1 is ongoing, whereas the ATV trail disturbance at HL ended during the early to mid 2000s [13]. No species were shared between HL and BM1 nor GH6 and BM1, while three species were shared between HL and GH6. This would indicate that despite a greater geographic distance, the fungal community present at HL is more similar to GH6 then it is to BM1, yet HL still remains less diverse than GH6. These findings are likely due to the differences in plant communities present at the three sampled sites. The HL site hosts a wide variety of herbaceous and woody plants that were not present at BM1 or GH6 (Supplemental Table S2). This difference in plant community could account for the unique fungi at HL, while the recovered state of the HL bog could explain the species shared between HL and GH6.

Despite all of the fungi being new records for *G*. *peckii*, the majority of the fungi found are known plant pathogens or saprobes of either conifers, hardwoods, or herbaceous plant hosts [24,25,26,27,28]. The encroachment of woody shrubs and competition with other herbaceous plants contributes to the population loss of *G*. *peckii* and was observed to varying degrees at all sites sampled (Supplemental Table S2). Woody plant species (e.g., huckleberry (*Gaylussacia baccata*), Labrador tea (*Rhododendron groenlandicum*), lambkill (*Kalmia angustifolia*), bog laurel (*Kalmia polifolia),* sweet gale (*Myrica gale*), and saplings of black spruce (*Picea mariana*) and speckled alder (*Alnus incana* ssp. *rugosa*) were common to all or most of our sampled sites (Supplemental Table S2). The fungi observed in *G*. *peckii* are potentially pathogenic to these surrounding woody plant species indicating that *G*. *peckii* may serve as a reservoir of numerous latent fungal pathogens, while further indicating that the fungi may not be pathogenic for the duration of their life cycles or that they may only be pathogenic to specific species in this bog ecosystem [3,29,30,31]. As pathogenicity was not documented for *G*. *peckii*, this suggests that these fungi can be pathogenic to other plant species in the surrounding environment, while they are non-pathogenic towards *G. peckii*. From a conservation perspective, *G*. *peckii* may host pathogens of its competitor plant species [30,32].

Due to sampling differences between months, it was not possible to determine the effect of time on observed fungal species richness. This said, previous studies have found that the foliar fungal diversity of plants in temperate to boreal climates can change with season and appears to peak between July and August [29,33]. This would suggest that in June, we likely sampled just prior to fungal peak, and in the future, it would be advantageous to sample over a longer time period to see if additional endophytic fungi might be uncovered. In the future we recommend a metagenomics approach to capture not only the culturable but also the unculturable fungal species richness present. However, a strength of our approach is the availability now of isolated fungi of interest for future research and use in plant propagation and restoration studies.

### 3.2. Known Ecologies of Sordariomycetes Recovered from Geum peckii

Of the 51 fungal taxa observed during our study, the majority (59%) were Sordariomycetes. Previous studies have found that foliar fungal endophytes are typically comprised of plant pathogens, endophytes, and latent saprobes from Sordariomycetes (most often isolated from woody material), as well as Dothideomycetes that are isolated from herbaceous material [32,34]. Saprophytic fungi are responsible for the decomposition of plant litter, which rapidly accumulates in bog habitats in temperate climates, and is broken down and incorporated into the substrate [34,35,36]. Sordariomycetes produce an array of enzymes capable of degrading cellulose, lignocellulose and hemicellulose present in woody plant cells [37]. Along Digby Neck, the bog soils are comprised of peat and mineral soil, and degradation of plant litter by microbes (including fungi) allows for the cycling of bioactive nutrients to occur [34,35,36]. Of the Sordariomycetes observed, 16 of the species were from the fungal family Gnomoniaceae. Fungi from this family typically occur on or in plant leaves and have been documented globally [26,27,38].

*Gnomoniopsis* species have narrow host ranges and are often pathogens of *Rosaceae* [38]. The species herein observed from this genus, *G*. *idaeicola*, *G*. *occulta*, and *G*. *macounii*, have been reported as only occurring within *Rubus* sp., *Potentilla* sp., and *Spirea* sp., respectively. We can now report that *G*. *peckii* is another *Rosaceae* host for these species. *Rubus* sp., *Potentilla* sp., and *Spirea* sp. were documented at most of our sites, though not always within the immediate vicinity of the *G*. *peckii* plants we sampled (see Supplementary Table S2).

Similarly, four previously described *Ophiognomonia* species known from *Picea rubens* (*O*. *acadiensis*) (detected at all sites other than the highly impacted site in our study), or from *Betula* and *Alnus* sp. (*O*. *alni-viridis*, *O*. *intermedia*) (located at three of our sites) and *O*. *ischnostyla* were reported by our study [27,38,39,40]. *Ophiognomonia* species were also found during our study. The finding of these species in *G*. *peckii* is unsurprising, as this plant is a member of the *Rosaceae* family, of which *Ophiognomonia* are known pathogens [27]. To fully understand the ecology of this fungal genus expanded host sampling is needed, to determine if the host range of this species is restricted to the *Rosaceae* [27,38]. *Asteroma alneum* was found at all our sites and all habitat types except GH4 and is a known pathogen of alder (*Alnus* spp.) [41]; *Alnus incana* ssp. *rugosa* was present at all our sites.

Of special note was the species *Cryptodiaporthe aubertii*, which was found as a *G*. *peckii* foliar endophyte at all sites. Intriguingly, this species has also been reported from the foliar tissues of *G*. *peckii* grown from seed in sterile tissue culture prior to ex situ planting in the Digby Neck bog habitat, where no *G*. *peckii* have previously been observed [15]. This fungus is a known endophyte and is possibly a latent saprophyte of Sweet Gale (*Myrica gale*), collected in bogs in Sweden and Russia [42,43]. *Plagiostoma lugubre* was also found at all sites and is a known endophyte and latent saprophyte with a wide range of host plants throughout the northern hemisphere [44]. *Diaporthe* sp. 4 was observed at all sites except CRN, and members of this genus have broad plant associations in temperate climates, where they have been reported as endophytes and minor plant pathogens [45]. Some species prefer damp habitats and are quick to colonise plant tissues, outcompeting other species [46,47].

Another commonly encountered endophyte was the hyphomycete *Microacsospora* sp., found at four sites (BM1, HL, GH4, GH6). Fungi in this genus may be coprophilous, with some species known from rotting plant matter and soil [48].

Fourteen fungi from the classes Leotiomycetes and Eurotiomycetes were isolated during our study, 11 of which were only found at a single site. Leotiomycetes are often found as plant endophytes and pathogens, showing low host specificity, and often co-occurring between hosts [49]. In the case of the coastal bog environment inhabited by *G*. *peckii*, Leotiomycete species have previously been reported from *Rhododendron* sp., *Vaccinium* sp. (*Coleophoma* sp., *Godronia* sp., and *Phacidium* sp.) [29,50,51], *Chamaedaphne calyculata*, *Andromeda polifolia*, *Kalmia angustifolia*, *Alnus* sp., *Betula* sp. (*Godronia* sp.) [29,52], and *Abies balsamea* (*Mollisia melaleuca*) [53]. The aquatic hyphomycetes *Varicosporium elodeae*, and *Articulospora* sp. have been isolated from the soil and roots of plants including *Picea glauca* [54,55]. These plant species were common to the sites that were sampled for this study. Eurotiomycetes are commonly saprobes [56].

## 4. Materials and Methods

### 4.1. Field Collection

Whole young healthy leaves were collected from living *Geum peckii* plants in the field under a scientific collecting permit from the Nova Scotia Department of Natural Resources which allowed each plant to be sampled only once. Specifically, tissues came from populations at Harris Lake (Digby Neck) and Brier Island (Digby County, NS, Canada). Five sites were sampled in 2015 (Figure 1 insets I–A and I–B): three on 12 June (BM1, GH6, and CRN) and 13 July (BM1, GH6, and CRN), as well as an additional two (GH4) on 13 July and (HL) 29 July. The scientific permit issued by NSDNR allowed site visits in June and July only, and limited which sites could be visited each month. The five sites sampled encompassed differing degrees of human impact from the pristine GH6 site (no known human impact) to the highly impacted BM1 site (impacted by bog draining, prior attempted use for agriculture crops, and the presence of a large Herring gull colony), to the three moderately impacted CRN, GH4, HL sites, which were characterised by past ATV trails across the sites and former sheep pasture [13]. Figure 1d provides example photographs of site conditions. For each month, at each site, the youngest healthy leaf, regardless of size, was collected from five living *Geum peckii* rosettes that were a minimum of 10 m apart. The tissue material collected from each plant sampled was placed into individual sterile plastic resealable bags. The youngest leaf was determined by the most recent petiole to emerge from the rosette. Leaves were then transported on ice to the laboratory and stored at 4 °C. Additionally, the dominant plant species observed within a two-meter radius of each sampled rosette were recorded (see Supplementary Table S2).

### 4.2. Endophyte Culturing and Molecular Identifications

Within 72 h of leaf collection, subsamples were taken using a sterile metal hole punch to create 1-cm-wide tissue discs. For each leaf, 8–24 tissue discs were taken, depending on the surface area of the leaf; discs were surface sterilised via: 30 s in 70% ethanol, immersion in 20% bleach for 7.5 min, a second 30 min rinse in 70% ethanol, and then a final 30 s rinse in autoclaved distilled H_2_O [57]. Tissue discs were plated onto two different growth media to maximise recovery of fungal endophyte species as media type is known to bias the fungal species which are isolated on it [3,32,58]. Half of the total number of tissue discs from each leaf were plated on 2% Malt Extract Agar (2% MEA), commonly used for fungal endophyte isolation, and half were plated on ½ Murashige and Skoog + Agar (½ MS + A) [58]. Petri dishes were sealed with Parafilm and incubated at 25 °C. Plates were observed daily, and fungi were isolated and transferred to 2% MEA to obtain axenic cultures of each species. Cultures were incubated as above (25 °C). Axenic cultures were preserved in the laboratory collection of AK Walker (Department of Biology, Acadia University, Wolfville, NS, Canada) in 2 mL sterile plastic microtubes containing 1 mL sterile distilled H_2_O stored at 4 °C and a duplicate tube containing 1 mL sterile 10% glycerol stored at −80 °C.

DNA extractions from axenic cultures were completed using a DNeasy UltraClean Microbial Kit (QIAGEN, Hilden, Germany) following the manufacturer’s protocol. PCR amplified fungal ITS rDNA (the accepted species-level barcode region for fungi, [59]) using primers ITS4 (5′-GCATCGATGAAGAACGCAGC-3′) and ITS5 (5′-TCCTCCGCTTATTGATATGC-3′) [60,61]. PCR reactions were carried out using 12.5 μL of 2× Ready PCR Mix (AMRESCO, LLC., Solon, Ohio), 1 μL each of forward and reverse primers (10 mM), 9.5 μL sterile distilled water, and 1μL of DNA template. Thermocycler settings were as follows: 95 °C for 3 min, then 35 cycles of 95 °C for 1 min, 56 °C for 45 s, and 72 °C for 90 s, followed by a final elongation step of 72 °C for 10 min. Agarose gel electrophoresis [10% gel *w/v* using 1 × TAE buffer at 95 volts for 30 min] was used to confirm positive DNA amplification. PCR products were sent for Sanger sequencing at Génome Québec Innovation Centre (McGill University, Montreal, Canada). DNA sequences were compared to sequences in the standard and TYPE sequence nucleotide BLAST database at GenBank (at NCBI (National Center for Biotechnology Information) using a ≥97% sequence identity threshold for making preliminary identifications, recognising additional genetic loci may require sequencing for species-level identifications in certain taxonomic groups (e.g., *Penicillium*, *Trichoderma*, *Fusarium*) [61]. DNA sequence data generated during this study are available on NCBI Genbank under accession numbers MW478644–MW478695.

### 4.3. Data Analyses

Photographs were taken on 13 July 2015 using a Canon Powershot SX240 HS camera; photo exposures were uniformly increased for each picture to allow better visualisation of the foliage/vegetation. Maps and the MCA analysis were constructed in RStudio Version 1.3.1093 [62] using R Version 4.0.3 [63]. Map making required the following additional R packages: ggplot2 [64], sf [65], tidyverse [66], ggspatial [67], and ggrepel [68]. Mapping data originates from https://gadm.org (accessed 31 October 2020). The MCA analysis required the following additional R packages: FactoMineR [69], factoextra [70], and ggplot2 [64]. To aid in making preliminary identifications based on ITS rDNA sequence data, sequence alignments 450–600 bp in length were generated and maximum likelihood phylogenetic trees were constructed in MEGAX^65^, each with 1000 bootstrap replications (see Supplemental Figures S1–S6) [71].

## 5. Conclusions

In summary, we present the first preliminary survey of foliar endophytic fungi from the rare and endangered Eastern Mountain Avens, *Geum peckii*. We documented 51 ascomycete fungi from the classes Sordariomycetes, Leotiomycetes, Dothideomycetes, and Eurotiomycetes. Endophytic fungal species richness changed with habitat degradation status, suggesting that this may influence the fungal assemblage present within leaves. A core fungal assemblage was documented for the first time in this plant host, from multiple sites throughout its Canadian range. Additionally, we present new host records for *Rosaceae* and make valuable linkages between host plants in the habitats and shared endophytes within *Geum peckii* that warrant further ecological investigation. By approaching plant conservation from a mycological perspective, we provide the first mycobiome assessment of *G*. *peckii*, an understudied component of the habitat. This new knowledge aids in ongoing conservation and propagation of this endangered plant species.

## Figures and Tables

**Table 1:** Fungi identified according to site, collection month, and isolation media. Data used in MCA indicated by an asterisk (*).

Taxa	Collection Site					No. Sites	Month		Media	
	GH6 *	HL *	CRN	GH4	BM1 *		June	July *	MEA	MS+A
Dothideomycetes										
*Alternaria* sp.	●					1	●		●	●
*Cladosporium* sp.		●				1		●	●	
*Didymocyrtis cladoniicola*					●	1		●	●	
*Neostagonospora elegiae*				●		1		●	●	
*Phaeosphaeria poagena*					●	1		●		●
*Ramularia* sp.					●	1		●	●	
*Stagonospora perfecta*					●	1	●			●
Eurotiomycetes										
*Penicillium* sp.	●					1		●		●
*Articulospora* sp.	●					1	●			●
*Coccomyces* sp.	●					1		●		●
Leotiomycetes										
*Godronia* sp.	●		●			2	●	●	●	●
*Helotiaceae* sp. 1	●					1		●		●
*Helotiaceae* sp. 2	●					1		●	●	●
*Lachnum virgineum*			●			1		●		●
*Mollisia melaleuca*	●					1		●	●	●
*Mollisia* sp.	●	●	●		●	4	●	●	●	●
*Phacidium* sp.	●					1	●		●	
*Phlyctema* sp.	●					1		●	●	●
*Phlyctema phoenicis*	●					1	●		●	
*Rhexocercosporidium* sp.			●	●		2		●	●	●
*Varicosporium elodeae*					●	1		●		●
Sordariomycetes										
*Apiognomonia hystrix*					●	1		●	●	
*Asteroma alneum*	●	●	●		●	4		●	●	●
*Colletotrichum* sp. 1					●	1	●		●	
*Colletotrichum* sp. 2				●		1		●	●	
*Cryptodiaporthe aubertii*	●	●	●	●	●	5	●	●	●	●
*Diaporthe* sp. 1				●		1		●		●
*Diaporthe* sp. 2				●		1		●	●	●
*Diaporthe* sp. 3			●			1		●		●
*Diaporthe* sp. 4	●	●		●	●	4	●	●	●	●
*Discula* sp.	●	●				2		●	●	●
*Fusarium* sp.					●	1		●	●	●
*Gaeumannomycella caricicola*		●	●			2	●	●		●
*Gnomoniaceae* sp. 1		●				1		●		●
*Gnomoniaceae* sp. 2	●					1		●	●	
*Gnomoniopsis idaeicola*					●	1		●	●	●
*Gnomoniopsis macounii*		●				1		●		●
*Gnomoniopsis occulta*				●		1		●		●
*Microacsospora* sp.	●	●		●	●	4	●	●	●	●
*Ophiognomonia acadiensis*	●	●	●	●		4		●	●	●
*Ophiognomonia alni-viridis*				●		1		●		●
*Ophiognomonia* aff. *gardiennetii*	●					1		●	●	●
*Ophiognomonia intermedia*	●	●		●		3		●	●	●
*Ophiognomonia ischnostyla*	●					1		●		●
*Ophiognomonia* sp. 1	●	●		●	●	4		●	●	●
*Ophiognomonia* sp. 2	●					1		●	●	
*Ophiognomonia* sp. 3		●				1		●		●
*Physalospora vaccinii*		●				1		●		●
*Plagiostoma lugubre*	●	●	●	●	●	5	●	●	●	●
*Plagiostoma* sp.	●					1		●		●
*Trichoderma* sp.	●					1	●			●
Totals =	27	16	10	14	16		14	44	31	40

## Data Availability

DNA sequence data are available at NCBI Genbank under accession numbers MW478644-MW478695. Additional data are available upon request from corresponding author.

## Supplementary Materials

The following is available online at https://www.mdpi.com/article/10.3390/plants10051026/s1, Table S1: Closest GenBank type sequence matches; Table S2: Plant species documented at study sites, Figures S1–S6: Maximum likelihood ITS rDNA trees generated in MegaX with 1000 bootstrap replications used to assign preliminary taxon identities.
