# Supplementary figures and images for: Foliar Endophytic Fungi from the Endangered Eastern Mountain Avens (Geum peckii, Rosaceae) in Canada
Source: Plants (Basel). 2021 May 20;10(5):1026. doi: 10.3390/plants10051026 (PMC8161203; doi:10.3390/plants10051026)

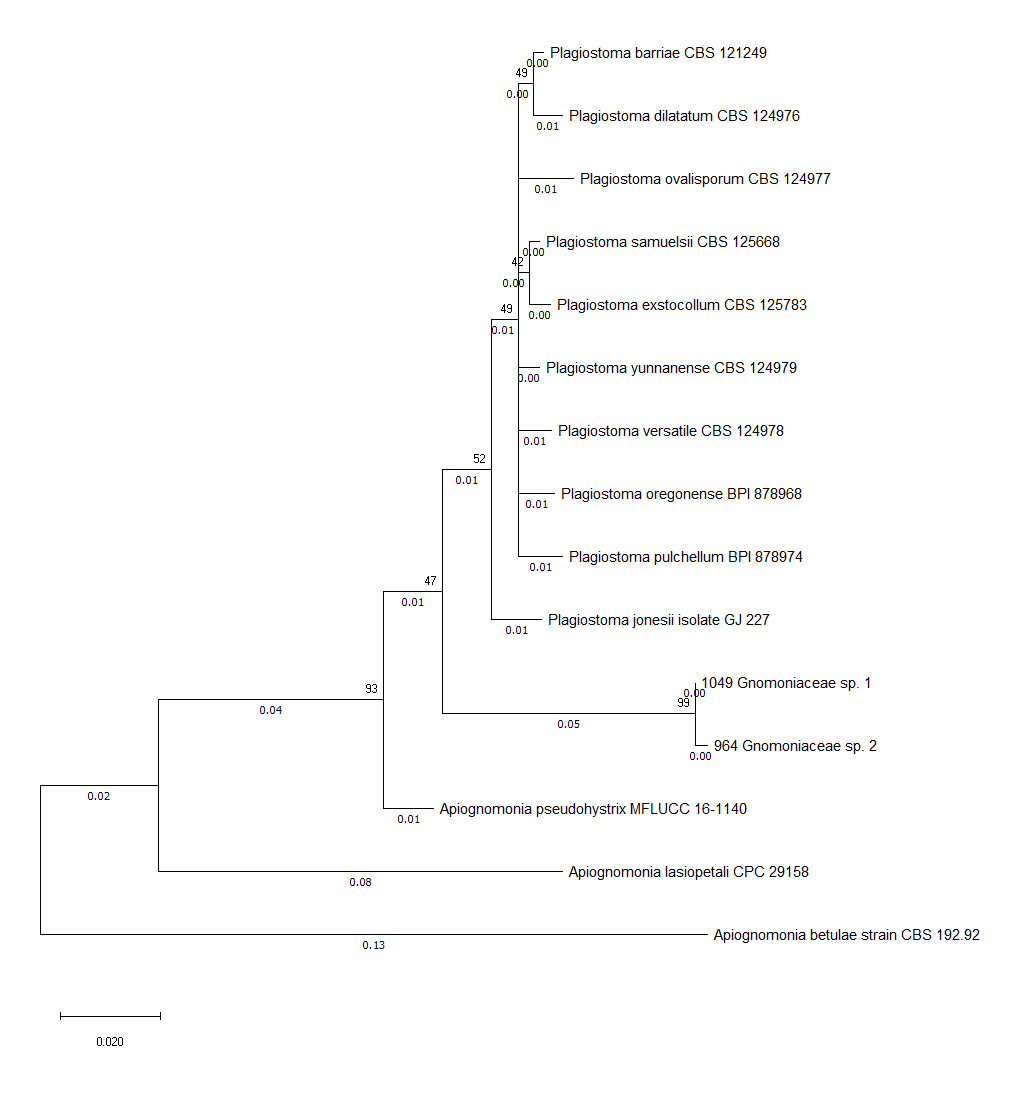

Supplement: Supplementary file 1 [file plants-10-01026-s001.zip › suppp/GnomoniaceaeMay17_revised_Suppl.png]

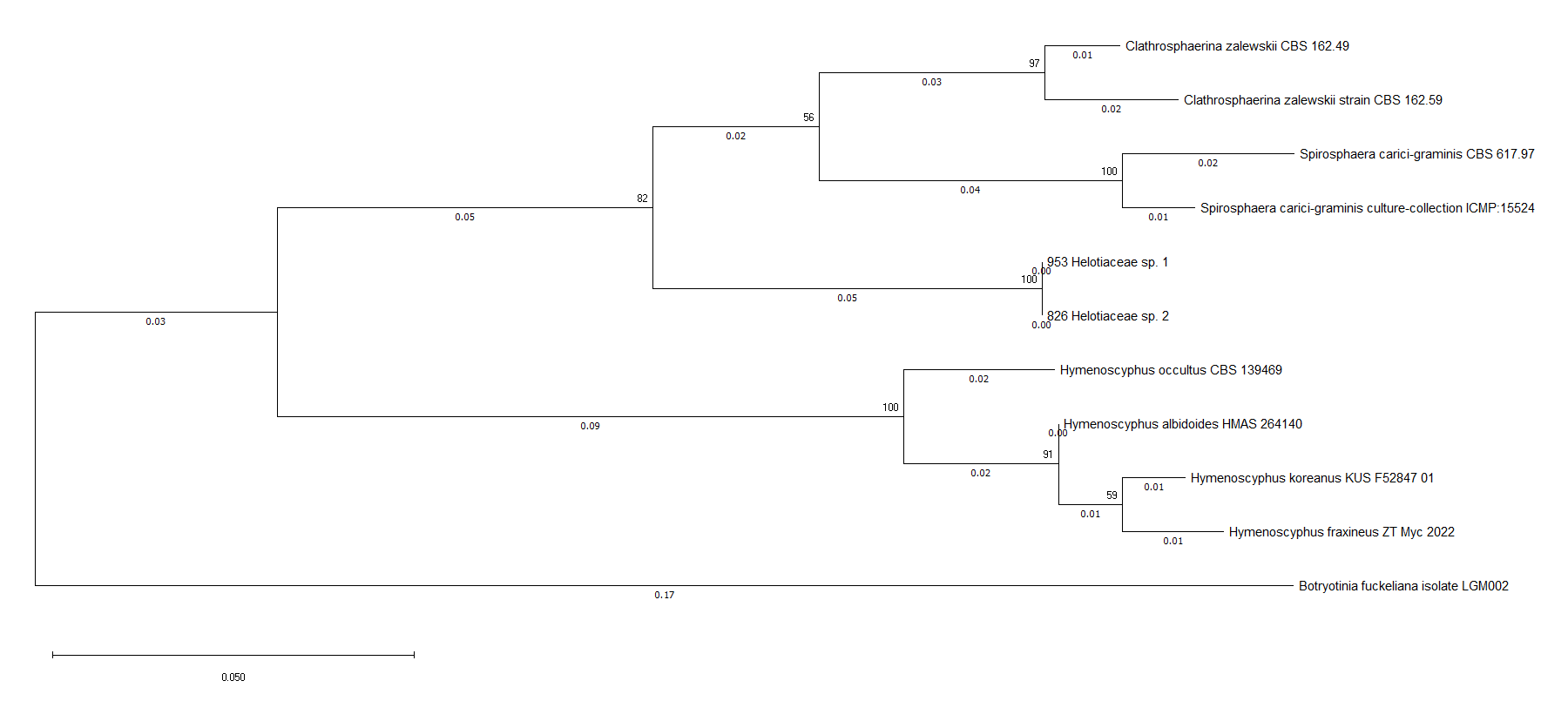

Supplement: Supplementary file 1 [file plants-10-01026-s001.zip › suppp/HelotiaceaeMay10.png]

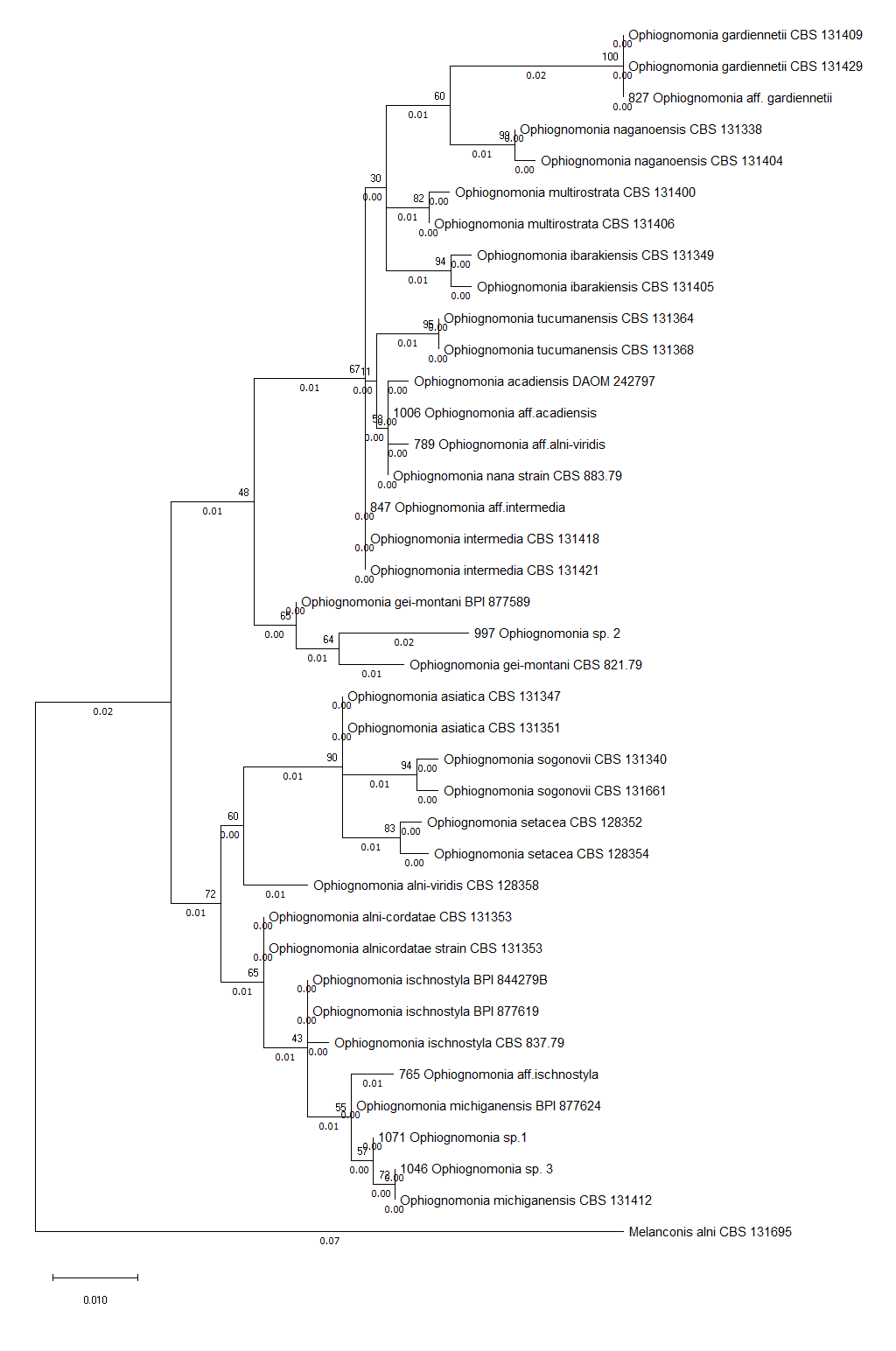

Supplement: Supplementary file 1 [file plants-10-01026-s001.zip › suppp/OphiognomoniaMay10.png]
